# Non-contrast free-breathing 3D cardiovascular magnetic resonance angiography using REACT (relaxation-enhanced angiography without contrast) compared to contrast-enhanced steady-state magnetic resonance angiography in complex pediatric congenital heart disease at 3T

**DOI:** 10.1186/s12968-022-00895-9

**Published:** 2022-11-17

**Authors:** Alexander Isaak, Narine Mesropyan, Christopher Hart, Shuo Zhang, Dmitrij Kravchenko, Christoph Endler, Christoph Katemann, Oliver Weber, Claus C. Pieper, Daniel Kuetting, Ulrike Attenberger, Darius Dabir, Julian A. Luetkens

**Affiliations:** 1 Department of Diagnostic and Interventional Radiology, University Hospital Bonn, Venusberg-Campus 1, 53127 Bonn, Germany; 2 Quantitative Imaging Lab Bonn (QILaB), Bonn, Germany; 3 Department of Pediatric Cardiology, University Hospital Bonn, Bonn, Germany; 4 Philips GmbH Market DACH, Hamburg, Germany

**Keywords:** Pediatric congenital heart disease, Cardiovascular magnetic resonance angiography, Non-contrast, Gadolinium-free, Relaxation-enhanced angiography without contrast

## Abstract

**Background:**

To evaluate the great vessels in young children with complex congenital heart disease (CHD) using non-contrast cardiovascular magnetic resonance angiography (CMRA) based on three-dimensional relaxation-enhanced angiography without contrast (REACT) in comparison to contrast-enhanced steady-state CMRA.

**Methods:**

In this retrospective study from April to July 2021, respiratory- and electrocardiogram-gated native REACT CMRA was compared to contrast-enhanced single-phase steady-state CMRA in children with CHD who underwent CMRA at 3T under deep sedation. Vascular assessment included image quality (1 = non-diagnostic, 5 = excellent), vessel diameter, and diagnostic findings. For statistical analysis, paired *t*-test, Pearson correlation, Bland–Altman analysis, Wilcoxon test, and intraclass correlation coefficients (ICC) were applied.

**Results:**

Thirty-six young children with complex CHD (median 4 years, interquartile range, 2–5; 20 males) were included. Native REACT CMRA was obtained successfully in all patients (mean scan time: 4:22 ± 1:44 min). For all vessels assessed, diameters correlated strongly between both methods (Pearson r = 0.99; bias = 0.04 ± 0.61 mm) with high interobserver reproducibility (ICC: 0.99 for both CMRAs). Native REACT CMRA demonstrated comparable overall image quality to contrast-enhanced CMRA (3.9 ± 1.0 vs. 3.8 ± 0.9, P = 0.018). With REACT CMRA, better image quality was obtained at the ascending aorta (4.8 ± 0.5 vs. 4.3 ± 0.8, P < 0.001), coronary roots (e.g., left: 4.1 ± 1.0 vs. 3.3 ± 1.1, P = 0.001), and inferior vena cava (4.6 ± 0.5 vs. 3.2 ± 0.8, P < 0.001). In all patients, additional vascular findings were assessed equally with native REACT CMRA and the contrast-enhanced reference standard (n = 6).

**Conclusion:**

In young children with complex CHD, REACT CMRA can provide gadolinium-free high image quality, accurate vascular measurements, and equivalent diagnostic quality compared to standard contrast-enhanced CMRA.

## Background

Cardiovascular magnetic resonance (CMR) is crucial for initial diagnosis, pre- and postoperative evaluation, and follow-up in children and adults with congenital heart disease (CHD) [1–3]. Cardiovascular magnetic resonance angiography (CMRA) provides radiation-free assessment of vascular structures and anomalies in children with CHD [4]. Contrast-enhanced CMRA techniques can generally be based on multiphase/time-resolved approaches and single-phase approaches with high spatial resolution using electrocardiogram (ECG) triggering and respiratory navigator gating [5, 6].

Although macrocyclic gadolinium-based contrast agents have an excellent safety profile, there are controversies regarding its retention in tissues after successive examinations [7]. However, the clinical significance is still unknown and there is currently no evidence of an associated toxicity [8, 9]. Nonetheless, strategies to reduce the gadolinium exposure are desirable, particularly in young children with CHD who undergo numerous CMR follow-up examinations during their lifetime. Gadolinium-free examinations also eliminate the risks of rare complications such as tissue necrosis after extravasation, allergic reactions, and nephrogenic systemic fibrosis. In addition, non-contrast examinations reduce examination costs and facilitate a faster and more time efficient clinical workflow.

Current non-contrast CMRA techniques for assessing the great vessels are typically based on balanced steady-state free precession (bSSFP) [10, 11]. Although they have shown promise in high-resolution imaging, the major drawback to off-resonance artifacts has limited their application, particularly at higher field strength like 3T or when covering a large field-of-view [12, 13]. Recently introduced flow-independent relaxation-enhanced angiography without contrast (REACT) technique employed a magnetization-prepared non-balanced dual-echo approach with 3D isotropic readout for native high-resolution CMRA [14, 15]. First studies in CHD using this technique has shown promising results in adults with predominantly corrected CHD at 1.5 T and other vascular territories [16–18]. Therefore, it may be a potential contrast-free alternative to visualize even more complex cardiovascular anatomies and much smaller vascular structures in a challenging cohort of young children with CHD at 3 T.

This study aimed to evaluate non-contrast REACT CMRA for assessment of the great vessels in young children with complex CHD in comparison to contrast-enhanced cardiac-gated steady-state CMRA as reference standard.

## Methods

### Study cohort

This retrospective study was approved by the local institutional review board that waived informed consent. From April to July 2021, consecutive young children with complex CHD, who had undergone CMR were identified. Patients aged < 10 years who had undergone both contrast-enhanced CMRA and non-contrast CMRA (for comparability in upcoming non-contrast follow-up examinations) under deep sedation were included for analysis. No exclusion criteria were applied regarding the type of CHD or previously performed surgical or interventional procedures.

### Cardiovascular magnetic resonance

All examinations were performed on a clinical whole-body 3T system (Ingenia Elition X, Philips Healthcare, Best, the Netherlands). For signal reception, a 16-channel body array coil (35 patients) or a 12-channel array coil (1 patient) with digital interface were used. The CMR protocol for CHD evaluation consisted of ECG gated bSSFP cine imaging in standard orientations, phase contrast velocity-encoded flow imaging of vessels of interest, and late gadolinium enhancement (LGE) in standard orientations. The CMR protocol was individually extended according to the type of CHD or specific cardiovascular abnormalities.

#### Non-contrast REACT CMRA

The imaging sequence applied was a 3D, magnetization-prepared, non-balanced, dual-echo acquisition with generalized Dixon for water and fat separation [14, 19]. For magnetization-preparation non-volume-selective T2-prep pulse and inversion recovery pulse with short inversion time were applied to suppress tissues with short to intermediate T1 and T2 relaxation times such as muscles, nerves, and internal organs. Signal of the native blood was enhanced due to its long T1 and T2 relaxation times, while residual fat signal was further removed with the help of Dixon in the obtained water images [20]. To minimize cardiac and respiratory motions, data acquisition was applied with prospective ECG (end-diastole) and respiratory gating (end-expiration). For imaging acceleration, a standard vendor implementation of the compressed sensing technology combined with parallel imaging, termed compressed SENSE (Philips Healthcare) [21, 22], was used with a factor of 5. In short, variable density sampling is used for data acquisition, while wavelet sparsifying transformation and L1-regularization are employed for online iterative reconstruction. REACT CMRA was acquired at the beginning of the scan protocol (before contrast injection) in all patients.

#### Contrast-enhanced CMRA

The high-resolution single-phase steady-state CMRA was acquired during a slow infusion (flow rate: 0.1–0.3 ml/s) of a gadolinium-based contrast agent at a dose of 0.1 mmol per kg of body weight (Gadobutrol, Gadovist, Bayer Healthcare, Berlin, Germany). Dual-echo Dixon readout was used to achieve fat removal in water images [20, 23]. Respiratory navigator gating for end-expiration and ECG triggering for end-diastole was applied for data acquisition. A compressed SENSE factor of 6 was used for imaging acceleration.

Both CMRA methods were applied in the coronal plane covering the chest. Imaging parameters are given in Table 1. Dixon-based water-only, fat-only, in-phase, and out-of-phase images were reconstructed and transferred for image analysis.

**Table 1:** Cardiovascular imaging parameters of native relaxation-enhanced angiography without contrast (REACT) and contrast-enhanced steady-state magnetic resonance angiography (CMRA) used in the present study

	Native REACT CMRA	Contrast-enhanced steady-state CMRA
Time of echo (ms)	1.42/2.8	1.93/3.4
Time of repetition (ms)	4.7	5.1
Orientation	Coronal	Coronal
Voxel size (mm³), acquired	1.39 × 1.40 × 1.40	1.19 × 1.20 × 2.40
Voxel size (mm³), reconstructed	0.45 × 0.45 × 0.70	0.69 × 0.69 × 1.20
Acquisition matrix (mm³)	216 × 214 × 125	252 × 249 × 80
Field of view (mm³)	300 × 300 × 88	300 × 300 × 96
Sampling of *k*-space	Cartesian	Cartesian
T2 prep/inversion delay time (ms)	50/10.8	No/320
Turbo field echo factor	31	19
Flip angle (°)	15	20
Compressed SENSE	Yes, factor 5	Yes, factor 6
Electrocardiogram gating	Yes	Yes
Number of heart phases	1 (single phase)	1 (single phase)
Respiratory gating	Yes, gating window (7 mm)	Yes, gating window (7 mm)

*CMRA* cardiovascular magnetic resonance angiography, *SENSE* sensitivity encoding

### Image analysis

Image quality assessment and vessel diameter measurements were performed independently by two radiologists in a blinded fashion for both CMRA methods (first reader: AI, 5 years of CMR experience; second reader: NM, 4 years of CMR experience) using a commercially available software (DeepUnity R20 XX, Dedalus HealthCare GmbH). Water-only images were primarily used for image analysis of both CMRA techniques (in-phase-images were additionally used if fat-water swapping artifacts were present).

#### Image quality

The following great vessels of the heart were defined as vessels of interest: ascending aorta, main pulmonary artery, left pulmonary artery, right pulmonary artery, left superior pulmonary vein, right superior pulmonary vein, superior vena cava, inferior vena cava. The origin of the right and left coronary artery were also evaluated. Image quality assessment was performed visually. Qualitative ratings were based on a five-point Likert scale defined as follows: (5) excellent = no artifacts, good vessel border delineation, (4) good = minimal artifacts, minimal vessel blurring, (3) intermediate = some artifacts, some vessel blurring, (2) poor = severe artifacts, severe vessel blurring, (1) non-diagnostic = vessels are not identifiable. The overall image quality score included ratings for all predefined vessels.

#### Vessel diameter measurements

For quantitative analysis, following vessels were analyzed: ascending aorta and descending aorta (at pulmonary bifurcation level), main pulmonary artery or conduit (midline between pulmonary valve and bifurcation), left and right pulmonary artery (or distal to the anastomotic area of Glenn shunt), left and right inferior, as well as superior pulmonary veins (1 cm distal to the atrial ostium or common trunk, respectively). Measurements were performed independently by both readers at the same predefined landmarks according to recent CMR guidelines for adults and children [24]. For each plane, the inner diameter was measured perpendicularly on multiplanar reconstructed images. Vessels with non-diagnostic quality were excluded from quantitative analysis.

#### Artifacts

The presence of artifacts compromising the great vessels was reviewed by both readers in consensus agreement. The evaluation included susceptibility artifacts (e.g., due to surgical or interventional material), flow artifacts (e.g., due to high and turbulent flow or valve insufficiency), and fat-water swap artifacts (Dixon method-specific signal swapping in calculated fat and water images) [16].

### Vascular findings

Most patients referred for CMR had complex cardiovascular anomalies, most of which were already known based on medical history and previous examinations (e.g., echocardiography, cardiac catheterization, or CMR). Final diagnosis of all vascular abnormalities and all accompanying clinically relevant findings based on CMRA was made in consensus by experienced, board-certified CMR readers (JAL with 10 years of CMR experience and CH with 17 years of CMR experience).

### Statistical analysis

Commercially available software (Prism, version 9.2, Graph-Pad Software, San Diego, California, USA) was used for statistical analysis. Data are presented as mean ± standard deviation or as absolute frequency. The Shapiro–Wilk test was applied to check for normal distribution of continuous data. Quantitative measurements between both CMRA methods were compared using the paired *t*-test, Pearson correlation, and Bland–Altman analysis. Differences in image quality ratings were tested using the Wilcoxon signed-rank test. The McNemar test was used to compare the frequency of occurring artifacts between both CMRA techniques. Intraclass correlation coefficients (ICC) were applied to analyze interobserver reproducibility [25].

## Results

### Patient characteristics

**Fig. 1:**
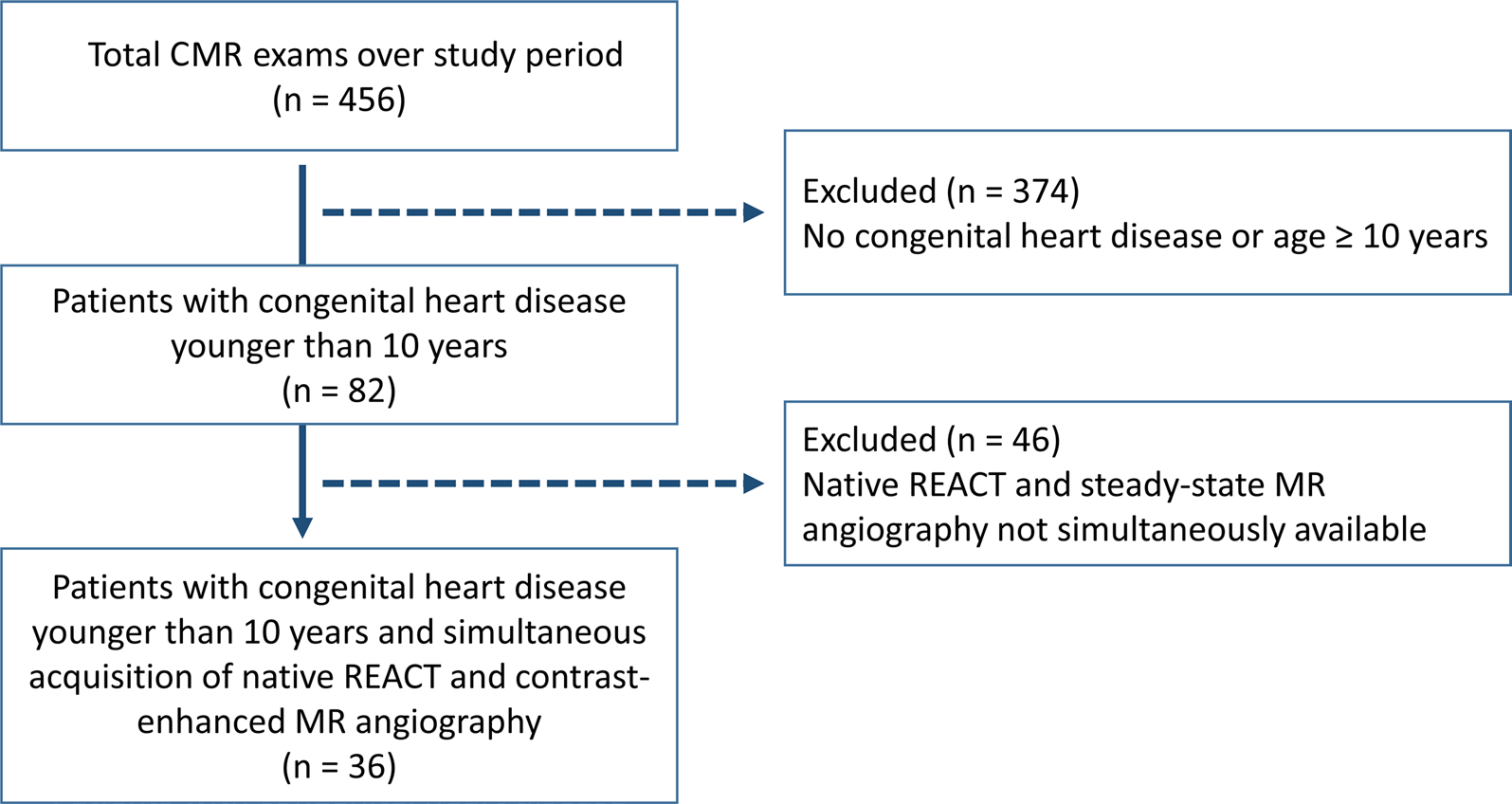
Study flow chart

Thirty-six pediatric patients with CHD were included in this study (median: 4 years, interquartile range, 2–5; weight: 17.4 ± 6.4 kg; 20 males, 56%) (Fig. 1). The age of 12/36 patients (33%) were ≤ 3 years, 18/36 patients (50%) were between 4 and 6 years, and 6/36 patients (17%) were between 7 and 9 years. Most patients had complex CHD with combinations of different heart defects (Table 2). The most common pathologies were tetralogy of Fallot (7/36, 19%) and hypoplastic left ventricle syndrome (7/36, 19%). Most patients (32/36, 89%) were referred for CMR for pre- or postsurgical evaluation. 15/36 patients (42%) had Glenn circulation. 8/36 patients (22%) had repair of tetralogy of Fallot. 32/36 patients (89%) were referred for pre- or post-surgical evaluation, and 4/36 patients (11%) with known CHD were referred for confirmation or exclusion of accompanying abnormalities during the clinical diagnostic workup. Further details on clinical and CMR characteristics are provided in Table 2.

**Table 2:** Clinical characteristics of included pediatric patients with congenital heart disease

Patient	Age (years)	Sex	Primary diagnosis	Indication for CMR
1	9	w	IAA, LVOTO, VSD, ASD	Evaluation of graft stenosis after Yasui procedure
2	6	m	TOF with PA, MAPCAs	Follow up after TOF repair (progressive right ventricular remodeling)
3	9	w	TOF, MAPCAs	Regular follow up after TOF repair
4	9	m	HLHS with AA, AVSD	Follow-up after Fontan procedure (Hypoxemia episodes)
5	4	m	TA, restrictive BVF, PVS, ASD	Evaluation before Fontan procedure
6	2	m	HLHC with MS and AS, AAH	Evaluation before Fontan procedure
7	6	m	TOF with PA	Follow up after TOF repair (decreasing physical capacity)
8	8	m	TOF	Follow up after TOF repair (low physical capacity)
9	9 months	w	DORV, PS, VSD	Progression of heart failure
10	5	m	TOF with PA, MAPCA	Evaluation after TOF repair and before surgery
11	2	w	DILV, DOLV, PA, VSD	Evaluation before Fontan procedure
12	2	m	TOF, ASD	Follow up after TOF repair (decreasing physical capacity)
13	5	m	HLHS	Evaluation before Fontan procedure
14	5	m	TOF, VSD	Follow up after TOF repair (moderate physical capacity)
15	7	m	Bicuspid aortic valve, AI	Critical AI
16	4	w	HLHS	Evaluation before Fontan procedure
17	4	m	DORV, VSD, ASD	Regular follow up after surgery/intervention
18	5	m	DILV, L-TGA, PS	Regular follow up after Fontan procedure
19	2	w	TA	Evaluation before Fontan procedure
20	5	m	HLHS (MA, AA), MAPCA	Evaluation before Fontan procedure
21	5	w	Suspected scimitar syndrome	Confirmation of PAPVC
22	2	w	HLHS (MA, AA), ASD	Evaluation before Fontan procedure
23	4	w	PA, MAPCAs	Evaluation before Fontan procedure
24	3	m	Unbalanced AVSD, ISTA	Evaluation before Fontan procedure
25	2	w	HLHS, VSD, ISTA	Evaluation before Fontan procedure
26	6	m	TOF, MAPCAs	Evaluation after TOF repair and before intervention
27	4	m	TAC, TS, VSD, ASD	Evaluation before Fontan procedure
28	7	m	Right atrial isomerism, AVSD, TAPVC, D-TGA	Regular follow up after Fontan procedure
29	3	w	DILV, L-TGA, ASD, ISTA	Evaluation before Fontan procedure
30	5	w	PA, VSD, ASD	Evaluation before Fontan procedure
31	2	w	TA, VSD, restrictive BVF, PVS	Evaluation before Fontan procedure
32	4	w	ASD, suspected PAPVC	Confirmation of diagnosis
33	4	w	Ebstein’s anomaly, PFO	Progression of heart failure
34	4	m	PA, VSD, ASD	Follow up after graft exchange
35	2	m	DORV, L-TGA, VSD, PFO	Evaluation before surgery (hypoxemia episodes)
36	1	w	Sinus venosus ASD, suspected PAPVC	Confirmation of diagnosis

*AAH* aortic arch hypoplasia, *AI* aortic insufficiency, *AS* aortic stenosis, *ASD* atrial septal defect, *AVSD* atrioventricular septal defect, *BVF* bulboventricular foramen, *DILV* double inlet left ventricle, *DOLV* double outlet left ventricle, *DORV* double outlet right ventricle, *D-TGA* dextro-transposition of the great arteries, *HLHC* hypoplastic left heart complex, *HLHS* hypoplastic left heart syndrome, *IAA* interrupted aortic arch, *ISTA* aortic isthmus stenosis, *L-TGA* levo-transposition of the great arteries, *LVOTO* left ventricular outflow tract obstruction, *MAPCA* main aortopulmonary collateral artery, *MS* mitral stenosis, *PA* pulmonary atresia, *PAPVC* partial anomalous pulmonary venous connection, *PFO* patent foramen ovale, *PS* pulmonary stenosis, *PVS* pulmonary valve stenosis, *TA* tricuspid atresia, *TAC* truncus arteriosus communis, *TAPVC* total anomalous pulmonary venous connection, *TOF* tetralogy of fallot, *TS* tricuspid stenosis, *VSD* ventricular septal defect

### Image acquisition

**Fig. 2:**
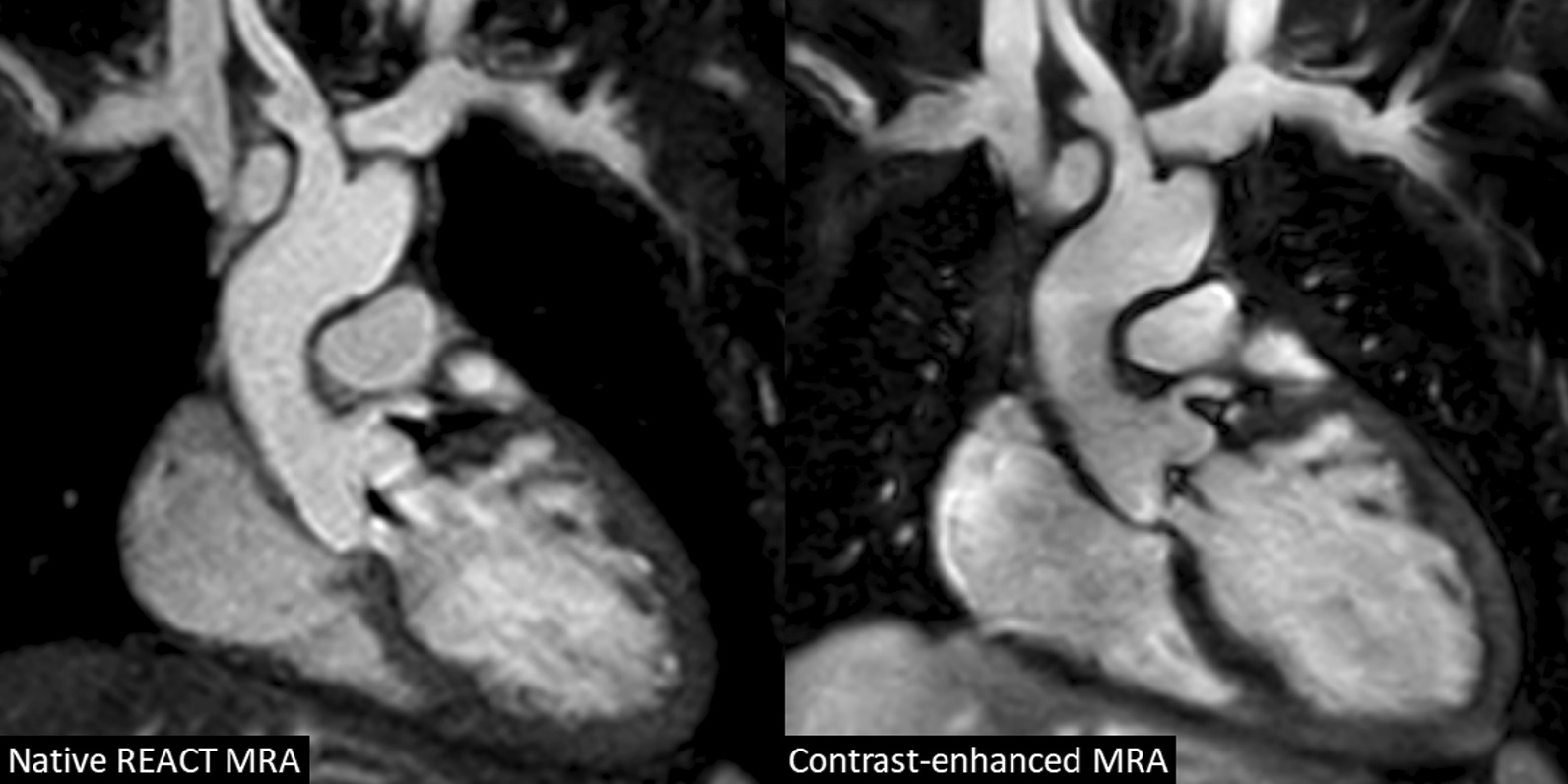
A 7-year-old boy with congenital aortic valve dysplasia (non-reformatted water-images in coronal view). The example demonstrates comparable image quality between native relaxation-enhanced angiography without contrast (REACT) and contrast-enhanced cardiovascular magnetic resonance angiography (CMRA) with excellent fat suppression, high contrast and good vessel border delineation. The left coronary origin is clearly delineated. The left atrial appendage lateral to the main pulmonary artery is partially covered. Note the aortic regurgitant jet due to diastolic acquisition

All patients had undergone CMR under deep sedation (propofol anesthesia). The mean total scan time was 4:22 ± 1:44 min for REACT CMRA and 1:51 ± 0:18 min for contrast-enhanced steady-state CMRA (P < 0.001). A representative imaging example of both CMRA methods is shown in Fig. 2.

### Image quality

**Fig. 3:**
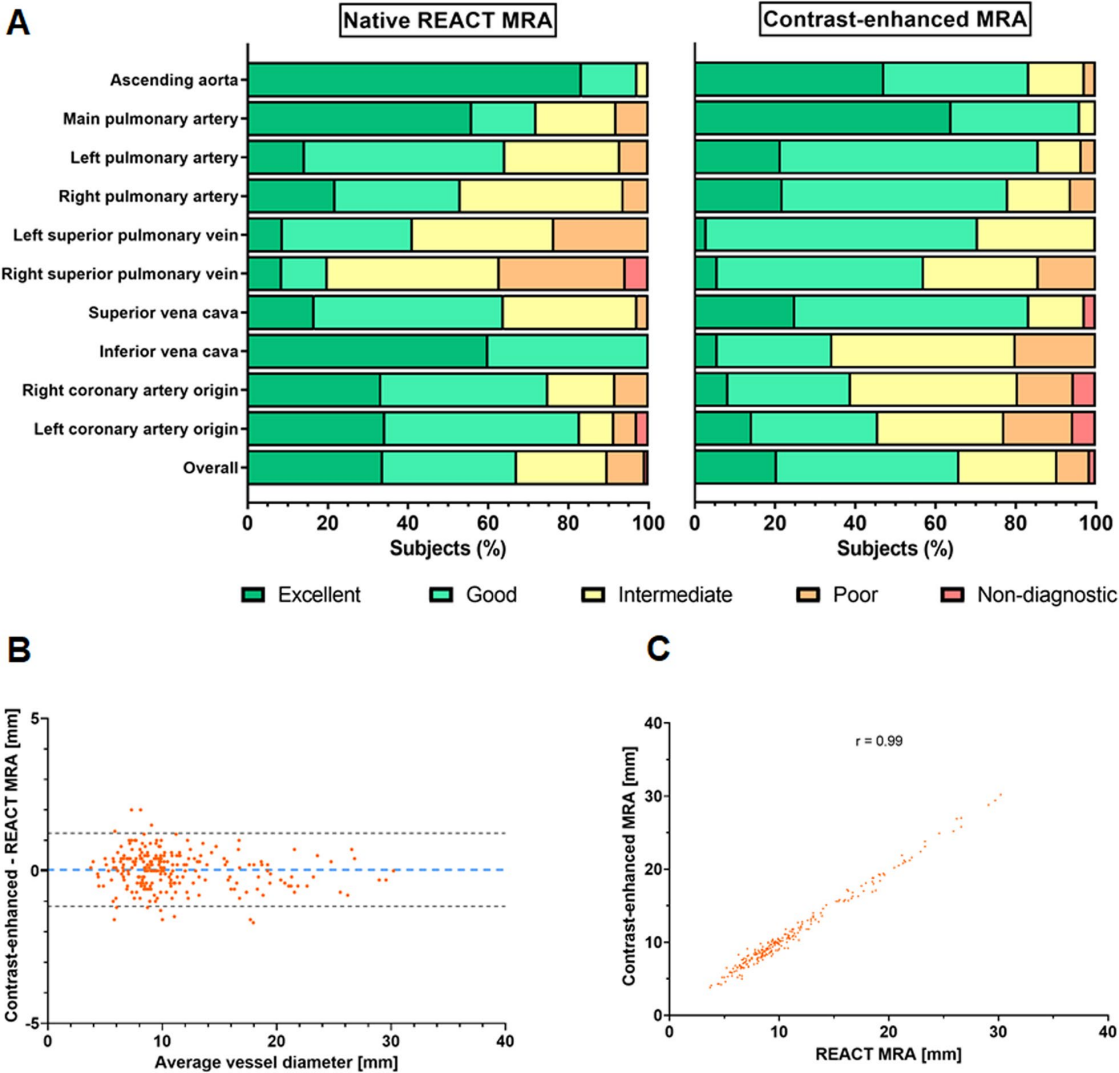
Image quality and vessel diameter measurements results. **A** Bar plots show image quality scores for native and contrast-enhanced CMRA. **B** Bland–Altman plot and, **C** scatter plot show correlation of all vessel diameter measurements between native and contrast-enhanced CMRA. Pearson’s correlation coefficient is given. *REACT* relaxation-enhanced angiography without contrast

**Fig. 4:**
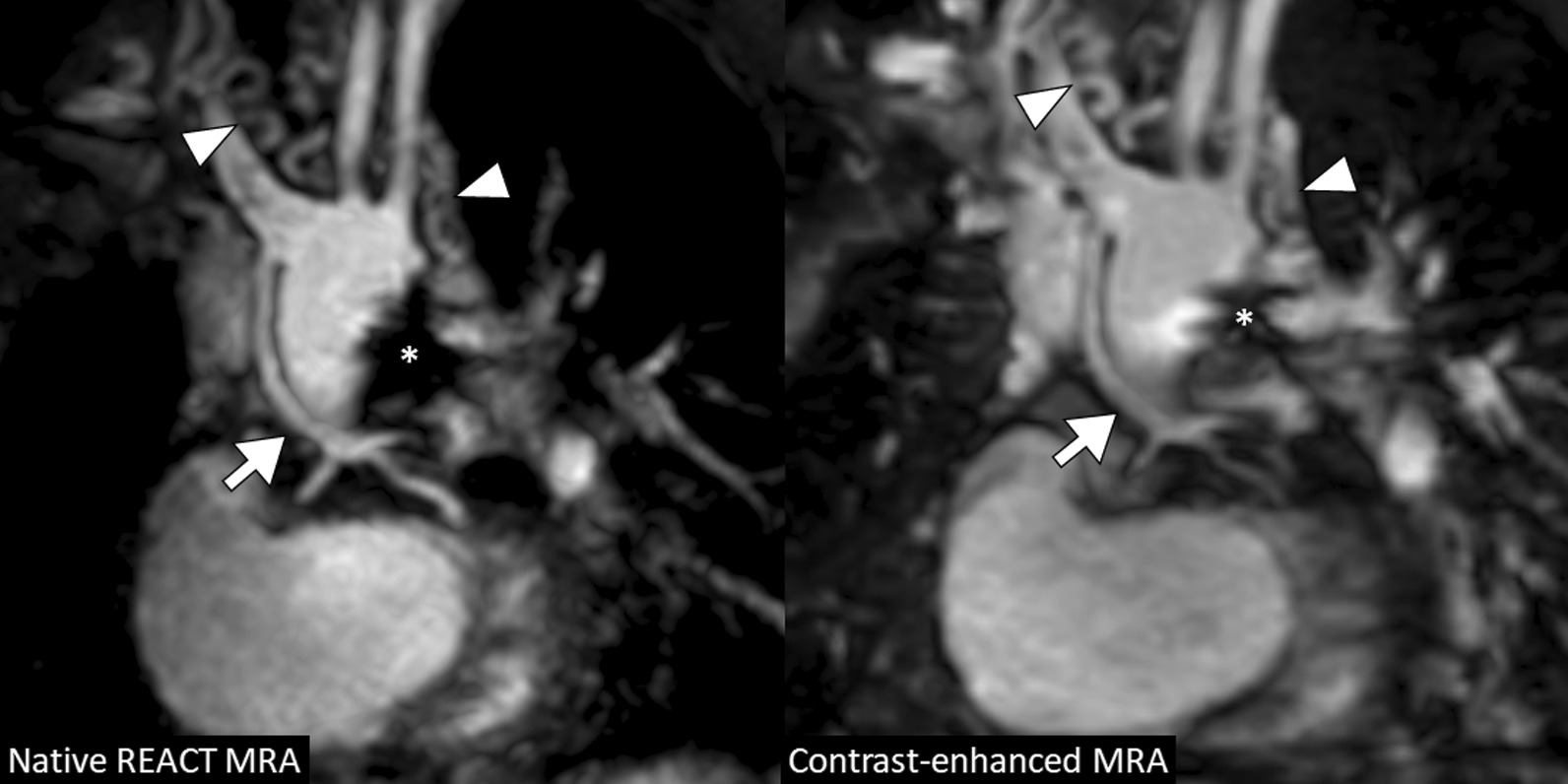
A 5-year-old boy with hypoplastic left heart syndrome (atresia of the mitral and aortic valves) and past medical history of several cardiac surgeries including Glenn procedure (multiplanar reformatted water-images in coronal view). Native and contrast-enhanced CMRA images demonstrate severely hypoplastic native ascending aorta that is connected to the brachiocephalic artery and shows distal trifurcation (arrow; note that the proximal coronary arteries are more clearly delineated on native images). Multiple small aorto-pulmonary collaterals with tortuous mediastinal course are visible (arrowheads). Susceptibility artifacts are present due to surgical stent material (asterisk). *REACT* relaxation-enhanced angiography without contrast

**Fig. 5:**
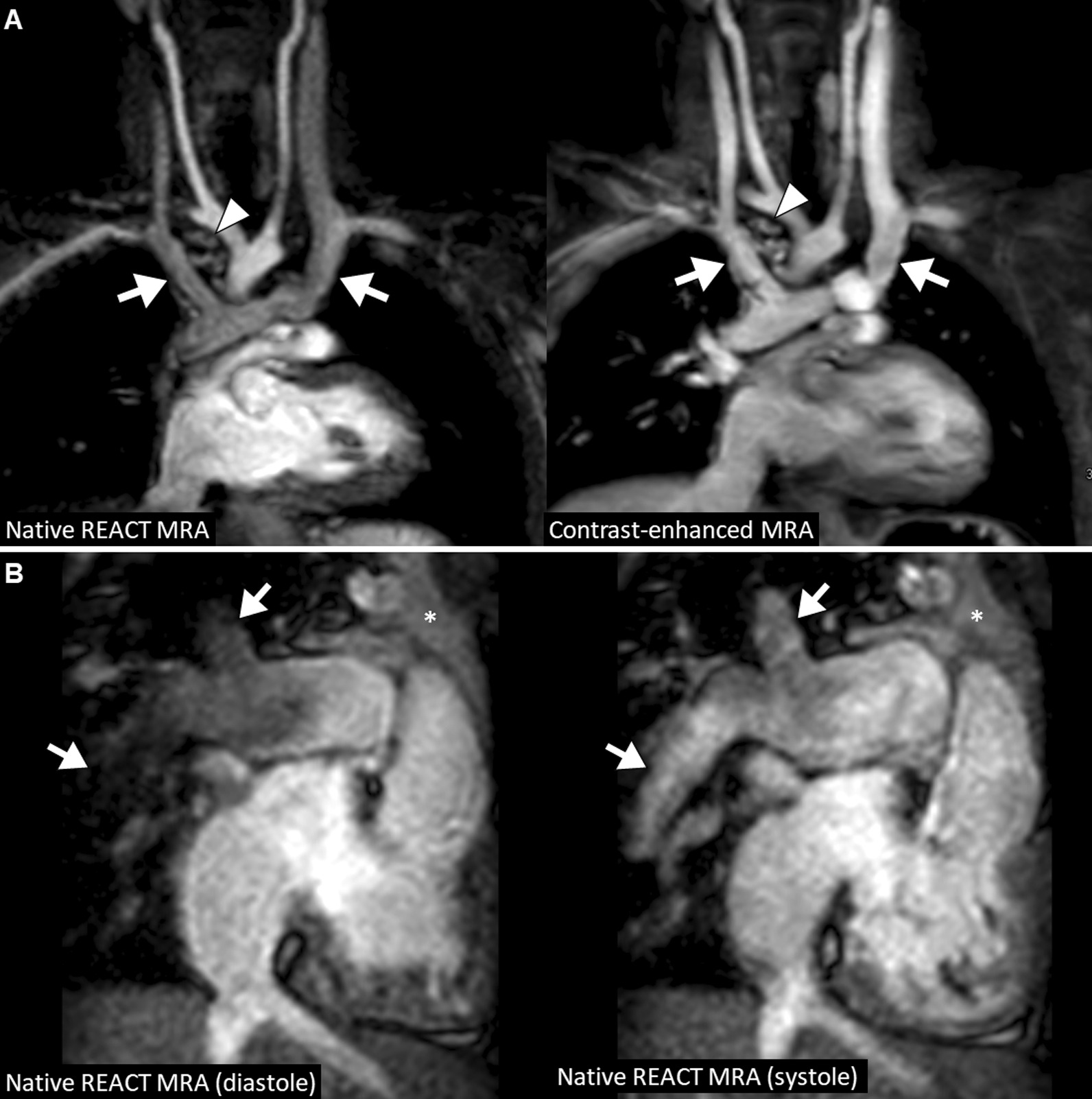
**A** 3-year-old boy with unbalanced atrioventricular septal defect (non-reformatted water-images in coronal view). Images shows lower blood signal intensity in the bilateral Glenn circulation on native compared to contrast-enhanced CMRA (arrows). These are presumably related to turbulent flow and differences in T1 an T2 relaxation times between venous and arterial blood. Small aorto-pulmonary collaterals are visible on both sequences (arrowhead). **B** A 5-year-old boy with congenitally corrected transposition of the great arteries and pulmonary trunk stenosis with dilatation of the right and left pulmonary arteries (multiplanar reformatted images in coronal-oblique view; out-of-phase images). Additionally performed systolic acquisition of native CMRA provides substantially reduced flow artifacts in the peripheral pulmonary arteries (arrows). Adjacent thymus (asterisk). *REACT* relaxation-enhanced angiography without contrast

In total, image quality was assessed by each reader for 330 vessels on REACT CMRA and contrast-enhanced steady-state CMRA, respectively (overall ratings, n = 660). 30 vessels (8%) were not assessable on both CMRA methods due to pronounced susceptibility artifacts or congenital or postoperative absence of vessel structures. The overall image quality score was slightly higher for REACT CMRA compared to steady-state CMRA (3.9 ± 1.0 vs. 3.8 ± 0.9, P = 0.018) (Fig. 3A). REACT CMRA achieved a higher image quality score compared to contrast-enhanced CMRA for the ascending aorta, the inferior vena cava, and the origin of the right and left coronary artery (Table 3). A clinical example with a variant origin of a severely hypoplastic native aorta with trifurcation of the coronary arteries is shown in Fig. 4. Image quality of pulmonary arteries was comparable between REACT and steady-state CMRA (Table 3). Image quality of REACT CMRA was slightly lower for the main pulmonary artery (Table 3), which was mainly contributed to flow artifacts due to severe pulmonary insufficiency in single patients. In patients with Glenn shunt (15/36, 42%), the cavopulmonary circulation had a lower blood signal intensity compared with the systemic circulation (Fig. 5A). The image quality of pulmonary veins was intermediate with significant better ratings for contrast-enhanced CMRA (Table 3). An example of a flow artifact is presented in Fig. 5B.

**Table 3:** Comparison of mean image quality scores between native relaxation-enhanced angiography without contrast (REACT) and contrast-enhanced magnetic resonance angiography (CMRA)

	Native REACT CMRA	Contrast-enhanced steady-state CMRA	*P* value
Ascending aorta	4.8 ± 0.5	4.3 ± 0.8	**< 0.001**
Main pulmonary artery	4.2 ± 1.0	4.6 ± 0.6	**0.031**
Left pulmonary artery	3.7 ± 0.8	4.0 ± 0.7	0.138
Right pulmonary artery	3.7 ± 0.9	3.9 ± 0.8	0.185
Left superior pulmonary vein	3.3 ± 0.9	3.7 ± 0.5	**0.005**
Right superior pulmonary vein	2.9 ± 1.0	3.5 ± 0.8	**0.003**
Superior vena cava	3.8 ± 0.8	4.0 ± 0.8	0.095
Inferior vena cava	4.6 ± 0.5	3.2 ± 0.8	**< 0.001**
Right coronary artery origin	4.0 ± 0.9	3.2 ± 1.0	**< 0.001**
Left coronary artery origin	4.1 ± 1.0	3.3 ± 1.1	**0.001**
Overall	3.9 ± 1.0	3.8 ± 0.9	**0.018**

The image quality was scored on a five-point scale (5 = excellent, 4 = good, 3 = intermediate, 2 = poor, 1 = non-diagnostic). Variables are given as mean ± standard deviation. P values refer to the Wilcoxon signed-rank test. Bold p-values indicate statistically significant results

### Vessel diameter measurement

Measurements of vessel diameter were performed for 285 landmarks by each reader (overall measurements, n = 570). 39 landmarks (12%) could not be assessed on both CMRA due to severe artifacts or aberrant cardiovascular anatomy. Vessel diameter measurements strongly correlated between both CMRA techniques and showed close intermethod agreement (Pearson r = 0.99; bias = 0.04 ± 0.61 mm, 95% limits of agreement: − 1.17 to 1.24 mm; Fig. 3B, C). Detailed results for vessel diameter measurements are given in Table 4. High ICCs indicated good interobserver reproducibility for the assessment of image quality (REACT: 0.87, contrast-enhanced CMRA: 0.79) and vessel diameter (REACT: 0.99, contrast-enhanced CMRA: 0.99) (Table 5).

**Table 4:** Measurements of vessel diameters with native relaxation-enhanced angiography without contrast (REACT) and contrast-enhanced magnetic resonance angiography (CMRA)

	Reader 1			Reader 2			
	Native REACT CMRA (mm)	Contrast-enhanced steady-state CMRA (mm)	P value	Native REACT CMRA (mm)		Contrast-enhanced steady-state CMRA (mm)	P value
Ascending aorta	19.2 ± 5.0	19.1 ± 4.9	0.174	19.2 ± 5.1		19.1 ± 4.8	0.253
Descending aorta	9.2 ± 1.4	9.1 ± 1.2	0.354	9.2 ± 1.3		9.2 ± 1.2	0.369
Main pulmonary artery/conduit	16.6 ± 5.6	16.5 ± 5.5	0.633	16.6 ± 5.5		16.6 ± 5.4	0.789
Right pulmonary artery	9.8 ± 2.5	9.8 ± 2.6	0.775	9.7 ± 2.5		9.7 ± 2.6	0.352
Left pulmonary artery	9.3 ± 3.4	9.5 ± 3.4	0.231	9.4 ± 3.2		9.5 ± 3.3	0.193
Right superior pulmonary vein	9.0 ± 2.2	9.0 ± 2.2	0.779	9.0 ± 2.2		9.0 ± 2.2	0.976
Right inferior pulmonary vein	9.1 ± 2.1	9.4 ± 2.1	**0.009**	9.1 ± 2.0		9.3 ± 1.9	**0.017**
Left superior pulmonary vein	7.5 ± 1.7	7.4 ± 1.7	0.635	7.6 ± 1.6		7.4 ± 1.7	0.291
Left inferior pulmonary vein	8.3 ± 1.7	8.6 ± 1.6	**0.011**	8.2 ± 1.6		8.4 ± 1.5	**0.033**

Data is given as means ± standard deviations. P values refer to paired *t*-test. Bold p-values indicate statistically significant results

**Table 5:** Intraclass correlation coefficients (ICCs) for interobserver variability accounting for the assessment of image quality and vessel diameters on native relaxation-enhanced angiography without contrast (REACT) and contrast-enhanced magnetic resonance angiography (CMRA)

	Native REACT CMRA	Contrast-enhanced steady-state CMRA
Image quality		
Overall	0.871 (0.840, 0.896)	0.792 (0.740, 0.833)
Aorta	0.704 (0.425, 0.849)	0.873 (0.752, 0.935)
Pulmonary arteries	0.921 (0.877, 0.949)	0.719 (0.567, 0.818)
Pulmonary veins	0.814 (0.701, 0.885)	0.753 (0.586, 0.850)
Vena cava	0.800 (0.677, 0.876)	0.803 (0.672, 0.880)
Coronary origin	0.727 (0.563, 0.830)	0.809 (0.666, 0.887)
Vessel diameters (mm)		
Overall	0.996 (0.995, 0.997)	0.995 (0.994, 0.996)
Aorta	0.998 (0.997, 0.999)	0.997 (0.995, 0.998)
Pulmonary arteries	0.995 (0.993, 0.997)	0.996 (0.993, 0.997)
Pulmonary veins	0.970 (0.957, 0.979)	0.965 (0.950, 0.975)

Numbers in parentheses are the 95% confidence intervals

### Artifacts

**Fig. 6:**
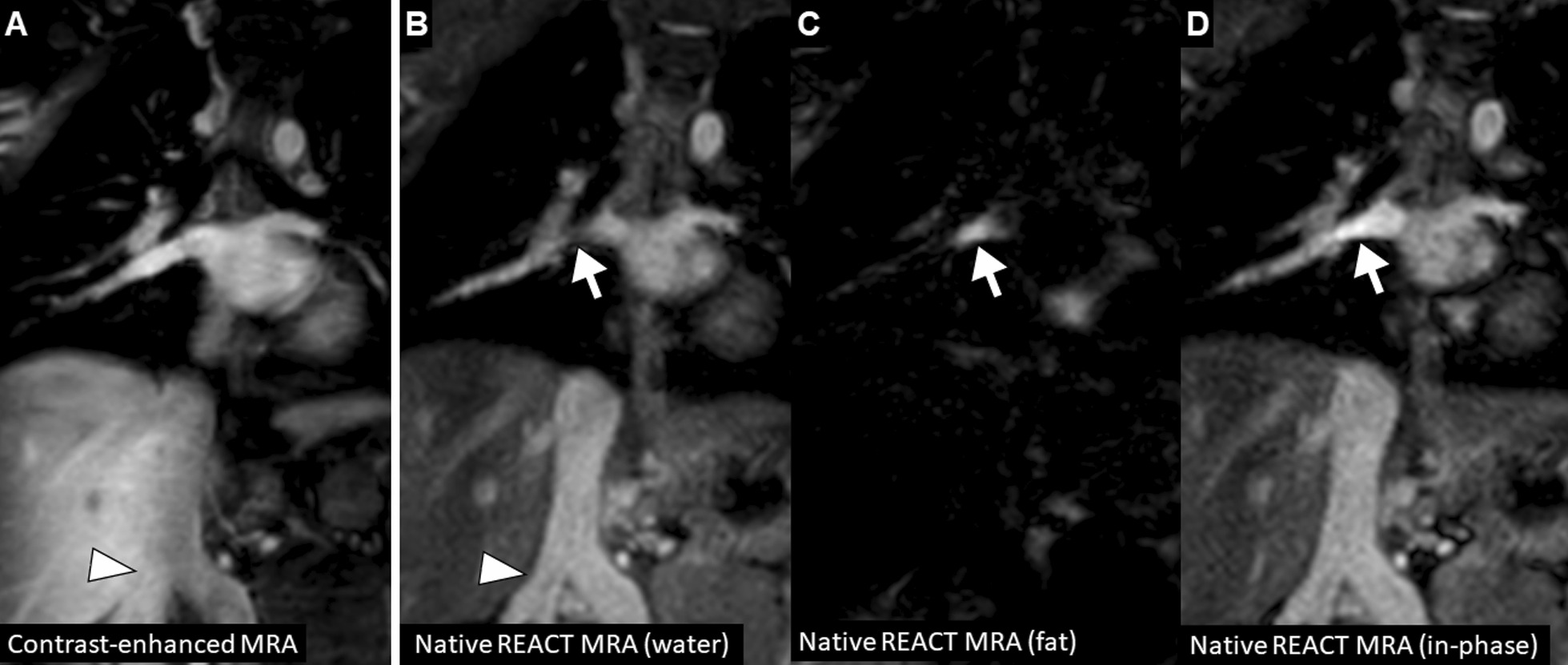
A 4-year-old boy with truncus arteriosus communis, ventricular septal defect, and dysplastic tricuspid valve (multiplanar reformatted images in coronal view show different image reconstructions derived from modified Dixon sequence). **A** Compared to the contrast-enhanced CMRA, a fat-water swapping artifact is seen on native REACT CMRA. **B** A hypointense signal is observed within the right inferior pulmonary vein (arrow) on native CMRA water-only image. **C** Fat-only image shows high signal in the same area indicating a fat-water signal swapping (arrow). **D** In this situation, the in- and out-of-phase images should also be generated, because they are usually not affected by this Dixon-specific artifact. Note the double inferior vena cava variant with better vessel delineation from the adjacent liver on native CMRA (arrowheads)

Susceptibility artifacts were found with comparable frequency on REACT and contrast-enhanced CMRA (14/36 [39%] vs. 15/36 patients [42%], P > 0.999). All of them were related to surgical or interventional material. Fat-water swapping artifacts occurred more frequently on REACT CMRA than on contrast-enhanced CMRA (12/36 patients [33%] vs. 6/36 patients [17%], P = 0.228). They were predominantly observed within the pulmonary veins (REACT CMRA: 10/36 patients [28%]; steady-state CMRA: 5/36 patients [14%]; P = 0.288). However, compensation for fat-water swapping was possible in all cases by reconstruction of in-phase and out-of-phase images (Fig. 6). Flow artifacts were only observed on REACT CMRA (8/36 [22%] vs. 0/36 [0%], P < 0.001) and affected primarily the pulmonary veins (4/36 patients [11%]) most likely related to high and turbulent flow and the pulmonary arteries (3/36 patients [8%]) due to severe pulmonary insufficiency (Fig. 5B).

### Vascular findings

**Fig. 7:**
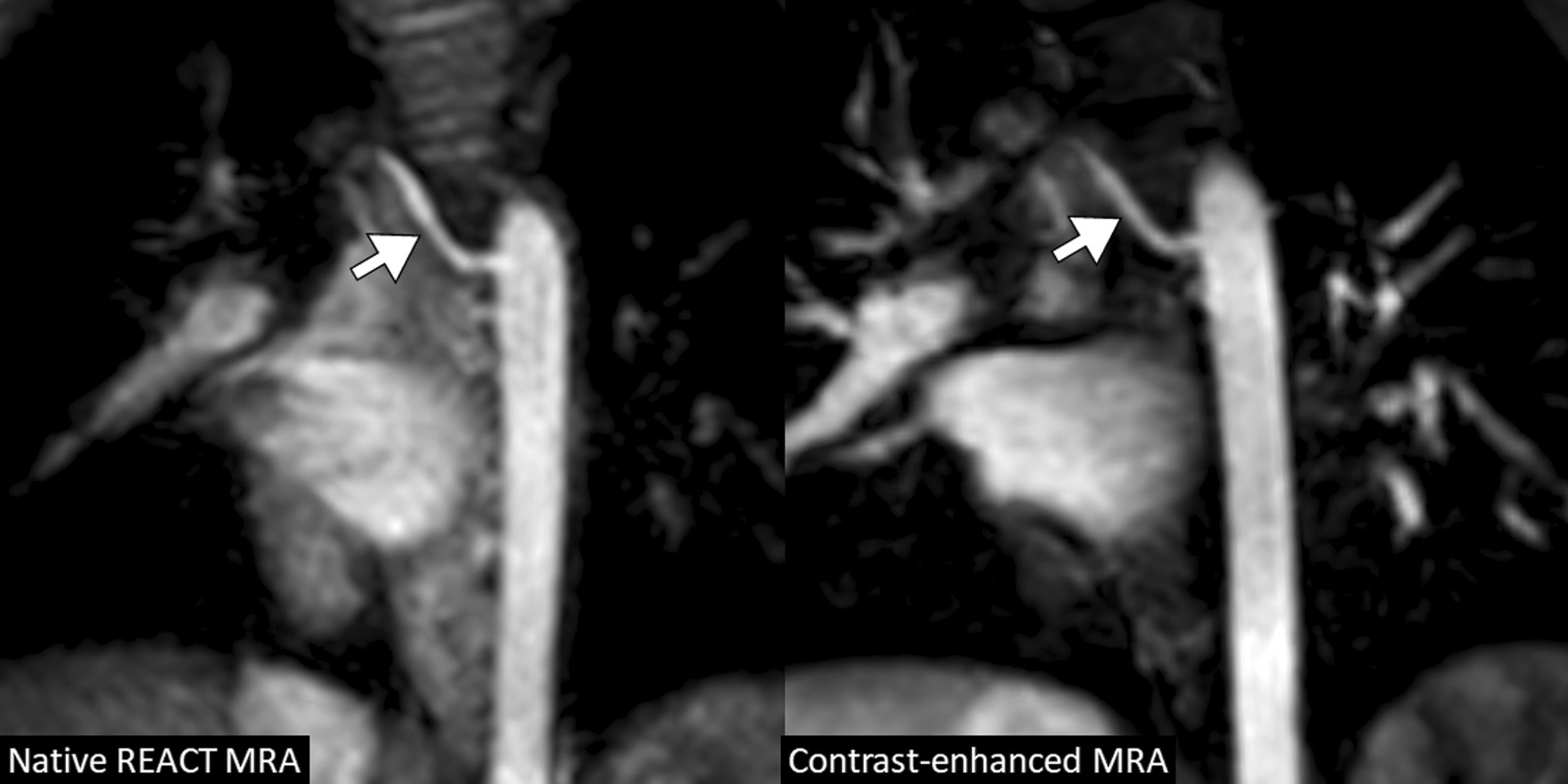
A 4-year-old boy with tricuspid atresia and pulmonary stenosis after Glenn procedure (multiplanar reformatted water images in coronal view). Native and contrast-enhanced CMRA images show dilated right bronchial artery originating from the descending aorta (arrow) indicating competing systemic supply to the lung by major aortopulmonary collateral arteries (MAPCAs). *REACT* relaxation-enhanced angiography without contrast

According to the specific indication for CMR, non-contrast REACT CMRA revealed the same vascular findings as contrast-enhanced CMRA in all patients. In patients who were referred for pre- or post-surgical evaluation (32/36, 89%), both CMRA sequences showed stable vascular conditions in most patients (27/32, 84%; one patient had a marked progressive dilatation of the proximal pulmonary arteries, two patients progressive new ectasia of the ascending aorta, one patient had Sano shunt obstruction and pulmonary vein stenosis, one patient had right ventricular outflow tract graft stenosis). Both CMRA techniques detected stable moderate proximal pulmonary artery stenosis in 7 of these 32 patients (22%). Eleven of these 32 patients (34%) showed stable aorto-pulmonary collaterals on both CMRA methods (mostly combinations of dilated bronchial artery branches, direct aortic branch, or indirect aortic branch via subclavian artery or internal mammary artery; Fig. 7). Four of these 32 patients (13%) had evidence of venous collateralization (predominantly via the dilated azygos system) detectable with both CMRA methods.

In patients referred for diagnosis confirmation or exclusion (4/36, 11%), there was no difference in final diagnosis between the two CMRA techniques. However, visualization of a partial anomalous pulmonary venous connection was limited in one patient on REACT CMRA compared to contrast-enhanced CMRA. In another patient a partial anomalous pulmonary venous connection could be excluded with both CMRA methods. Two patients with known CHD who were primarily referred for evaluation of progressive heart failure, did not present relevant vascular abnormalities on both CMRA methods.

**Fig. 8:**
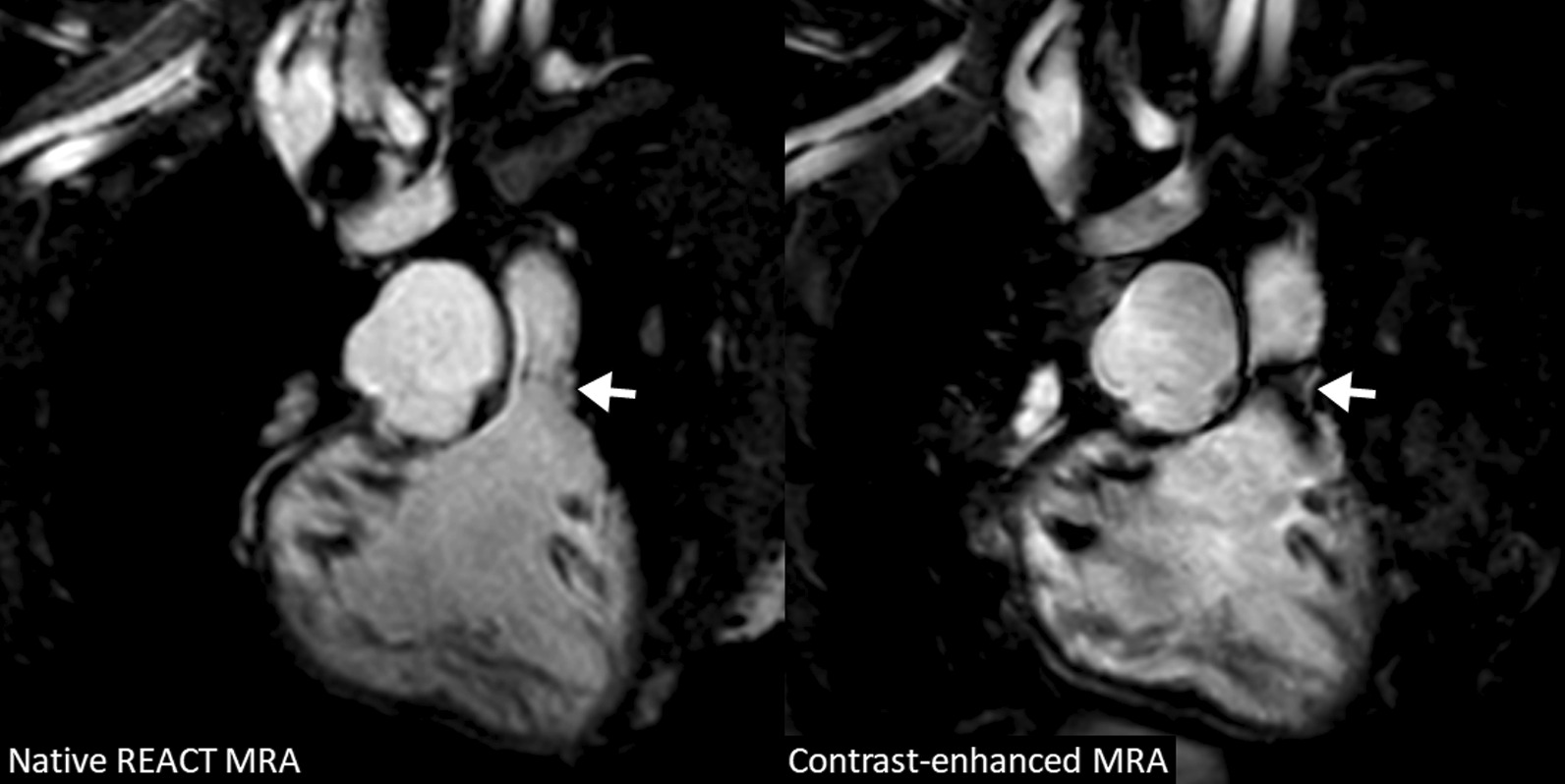
A 9-year-old girl with interrupted aortic arch and post operative situation (non-reformatted water images in coronal view). Native CMRA has less susceptibility artifacts at the pulmonary valve level (arrow) than contrast-enhanced CMRA and allows for accurate assessment of the right ventricular outflow tract dimension. *REACT* relaxation-enhanced angiography without contrast

In 3/36 patients (8%), the main pulmonary arteries could not be evaluated on REACT CMRA due to distinct flow artifacts caused by severe pulmonal insufficiency (corresponding phase contrast measurements revealed a regurgitation fraction of > 40% in these patients). However, in one patient stenosis of a right ventricular outflow tract graft was more clearly assessable on REACT CMRA compared to contrast-enhanced CMRA (Fig. 8). Furthermore, a coronary anomaly in one patient was only rudimentarily visualized on contrast-enhanced CMRA, but clearly delineated on REACT CMRA.

**Fig. 9:**
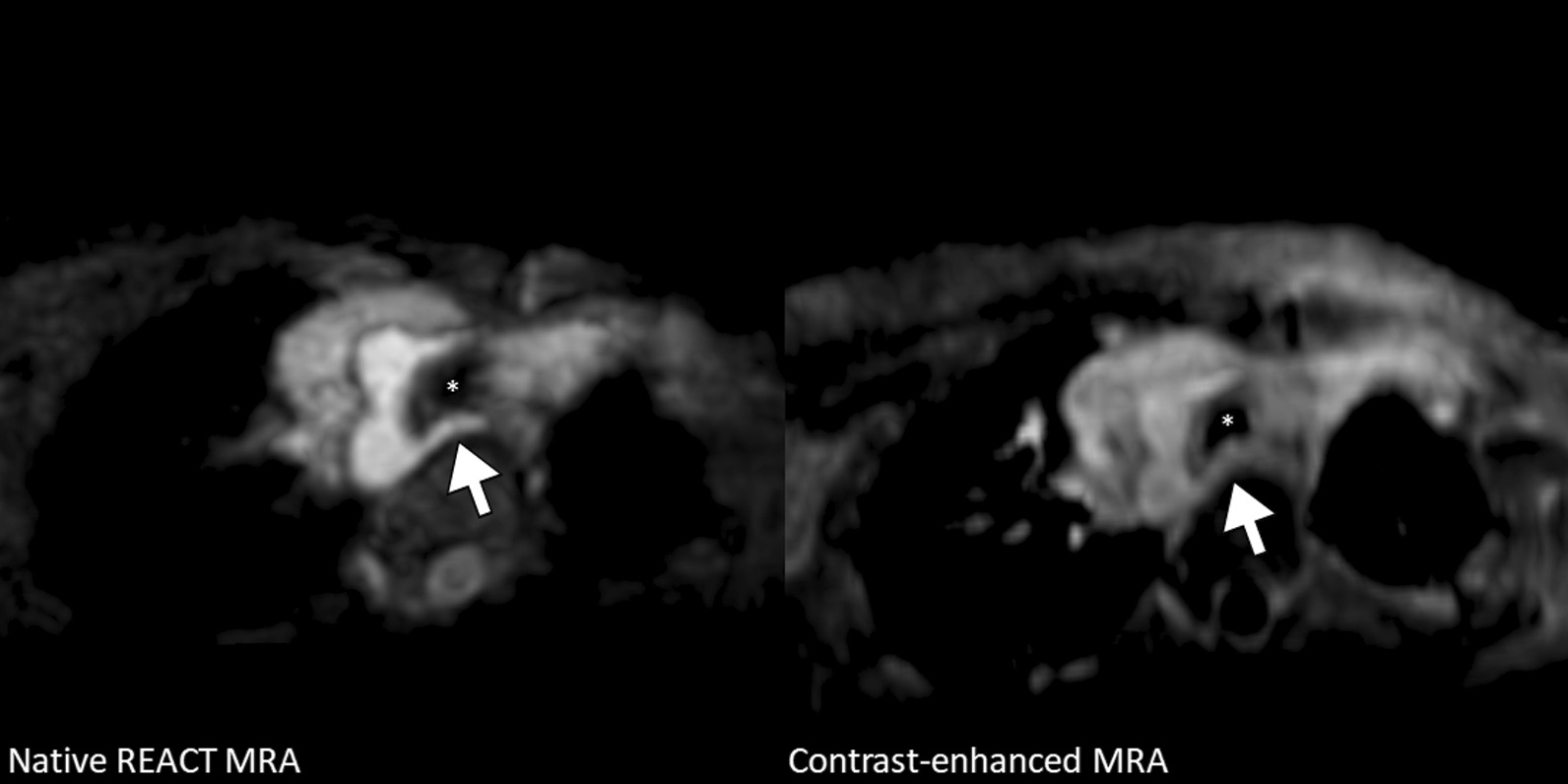
A 1-year-old girl with sinus venosus atrial septal defect (multiplanar reformatted water-only images in transversal view). Images show right-sided aortic arch with aberrant left subclavian artery without associated Kommerell diverticulum passing the trachea (asterisk) and the compressed esophagus posteriorly. Native CMRA provides better vessel contrast and delineation from adjacent structures, such as the anterolaterally located thymus, compared to contrast-enhanced CMRA.

Concomitantly, REACT and contrast-enhanced steady-state CMRA allowed for the visualization of accompanying findings with clinical relevance, like abnormalities of the supraaortic arteries (e.g., visualization of aberrant subclavian artery, Fig. 9) or the cervical veins (e.g., internal jugular vein occlusion).

## Discussion

This intraindividual comparison study presents a non-contrast high-resolution 3D isotropic CMRA that demonstrated high overall image quality and equivalent diagnostic findings in young children (median age: 4 years) with complex CHD, compared to contrast-enhanced steady-state CMRA. Improved image quality at the ascending aorta including the proximal coronary arteries and at the inferior vena cava underline that native REACT CMRA can be implemented for clinical use in CHD.

Due to the ongoing debate on CMR contrast deposit in the human body, efforts to reduce the use of gadolinium contrast media have been intensified over the recent years. So far there is no standard approach for gadolinium-free CMRA in children with complex CHD. To accurately assess vessel diameter and clearly visualize even small vessels and vascular connections in CHD, high spatial resolution cardiac and respiratory gated CMRA is often complementary performed to time resolved multiphase CMRA [23]. However, its acquisition requires additional contrast agent administration. Considering the young age of CHD patients, high-resolution non-contrast-enhanced techniques are strongly desirable in clinical practice but are often not applied owing to a lack of validation. Acquisition of the proposed REACT CMRA was successful in all patients with free breathing. Non-contrast REACT CMRA could be acquired early during the CMR examination, providing a three-dimensional overview of the entire cardiovascular anatomy and facilitating cine and flow imaging planning. The mean acquisition time for REACT CMRA was longer than contrast-enhanced CMRA. Considering the total scan duration of a typical CMR protocol for CHD, this was still within an acceptable range. Previous studies on native CMRA imaging in a pediatric CHD cohort reported longer mean scan times of approximately 7–10 min (max. 18 min) using a bSSFP whole-heart technique and conventional navigator [26, 27]. However, the majority of non-contrast-enhanced CMRA studies focused on coronary vessels rather than the great vessels and were not based on young pediatric CHD.

REACT-CMRA provided accurate and reliable measurements of vessel diameter comparable to contrast-enhanced CMRA. Although there was a small difference between both methods in the vessel diameter of the inferior pulmonary veins (probably related to impaired image quality due to flow artifacts on REACT CMRA), the difference was still within an acceptable range. The overall image quality score of REACT CMRA was comparable to contrast-enhanced CMRA. Major benefits in image quality were achieved at the ascending aorta including the proximal coronary arteries and at the inferior vena cava.

Excellent diagnostic quality of the ascending aorta on non-contrast REACT CMRA allowed for accurate detection of aortic ectasia at pre- or postsurgical follow-up (two patients). Because of the high prevalence in CHD, concomitant assessment of aberrant coronary anatomy is of particular value [28]. In clinical routine, acquisition of coronary whole heart imaging is often necessary in addition to thoracic contrast-enhanced CMRA [28], which extends examination time. Here, REACT CMRA could reduce examination time by combined assessment of the great vessels and coronary arteries. Furthermore, visualization and accurate measurements of the superior and inferior vena cava are important in patients with Glenn or Fontan circulation for pre- or postsurgical follow-up [29, 30]. REACT CMRA partially showed moderate image quality of the superior vena cava and pulmonary arteries due to Glenn or Fontan circulation (50% of patients) with lower venous blood signal and flow turbulences, but still reached adequate diagnostic quality.

The slightly impaired image quality for the pulmonary arteries and the intermediate image quality for the pulmonary veins were mainly contributed to artifacts caused by high and turbulent pulmonary flow (especially in patients with severe pulmonary insufficiency or high and turbulent vein flow during diastole). However, clinically relevant abnormalities such as dilatation or stenosis of the pulmonary arteries were adequately assessed compared to contrast-enhanced CMRA. Turbulent flow effects occurred particularly in highly pulsatile circulations like in patients with repaired tetralogy of Fallot. It is known from previous whole heart imaging studies that these effects can be reduced or even compensated by data acquisition in different cardiac phases [27]. Our results indicate that the unique and complex cardiovascular hemodynamics in young children with CHD are not generally comparable with an adolescent or adult cohort [16, 31, 32] and might require dedicated technical adjustments for non-contrast imaging due to pronounced flow-related effects.

As most patients in our cohort were referred for pre- or postsurgical follow-up with known CHD, most findings of vascular abnormalities were stable. However, all additional vascular findings diagnosed with contrast-enhanced CMRA could also be visualized with REACT-CMRA (e.g., progressive dilatation of the great vessels or graft/shunt stenosis). There was no difference in the detection of aorto-pulmonary collateral arteries or signs of venous collateralization between both CMRA techniques. In two patients (6%) the use of REACT CMRA was beneficial compared to contrast-enhanced CMRA (evaluation of graft stenosis and detection of a coronary anomaly). However, in three patients (8%) with severe pulmonary insufficiency, the main pulmonary arteries could not be adequately assessed on REACT CMRA because of distinct flow artifacts. In one patient (3%) a partial anomalous pulmonary venous connection was better depicted on contrast-enhanced CMRA.

CMR is particularly well suited for contrast reduction because it is usually based on double dose contrast administration owing to LGE imaging. The assessment of disease- or surgery-related myocardial fibrosis by LGE imaging may improve risk stratification, especially in grown-up patients with CHD [33–35]. As the concrete prognostic value of repeated LGE imaging in children is poorly studied, its acquisition on regular follow-up is not essential and currently depends on individual clinical decision making. In young patients with known and clinically stable CHD and no clinical indication for time-resolved CMRA or LGE imaging, a contrast-free protocol is reasonable, and could be complemented by the use of non-contrast REACT CMRA.

### Limitations

Our study has several limitations. The number of patients in the current study is relatively small. However, a wide range of complex CHD types with postoperative conditions were included, suggesting applicability in pre- and postoperative follow-up. Nevertheless, studies with larger patient cohorts are needed to provide subgroup analyses of different age groups and CHD types to further standardize the approach in different clinical settings. There might be an observer bias, as readers could not be blinded to the CMRA techniques for qualitative and quantitative analyses. In this study, only morphological vascular findings could be assessed with REACT and single-phase contrast-enhanced CMRA, but not functional findings detectable with cine and flow imaging or time-resolved CMRA. Signal and contrast to noise analysis were not performed due to different acceleration factors. Furthermore, comparison of REACT CMRA to other non-contrast techniques such as bSSFP-based or Quiescent-Interval Single Shot (QISS) CMRA was beyond the scope of this study. However, bSSFP CMRA is generally limited at 3T due to predominant off-resonance artifacts caused by field inhomogeneity.

## Conclusion

In conclusion, non-contrast REACT CMRA provides high image quality, accurate vascular measurements, and equivalent diagnostic certainty compared to high-resolution contrast-enhanced steady-state CMRA in a challenging cohort of young children with complex CHD. As part of a standard CMR protocol, REACT CMRA can enable gadolinium-free examinations without compromises in diagnostic quality for children with CHD undergoing pre- or postsurgical follow-up.

## Data Availability

The datasets generated and/or analyzed during the current study are not publicly available due data protection but are available from the corresponding author on reasonable request.

## Abbreviations

bSSFP: Balanced steady-state free precession
CHD: Congenital heart disease
CMR: Cardiovascular magnetic resonance
CMRA: Cardiovascular magnetic resonance angiography
ECG: Electrocardiogram
LGE: Late gadolinium enhancement
REACT: Relaxation-enhanced angiography without contrast

## References

[1] Triedman JK, Newburger JW (2016). Trends in congenital heart disease: the next decade. Circulation.

[2] Baumgartner H, de Backer J, Babu-Narayan SV, Budts W, Chessa M, Diller G-P (2021). 2020 ESC guidelines for the management of adult congenital heart disease. Eur Heart J.

[3] Sachdeva R, Valente AM, Armstrong AK, Cook SC, Han BK, Lopez L (2020). ACC/AHA/ASE/HRS/ISACHD/SCAI/SCCT/SCMR/SOPE 2020 appropriate use criteria for multimodality imaging during the follow-up care of patients with congenital heart disease: a report of the American College of Cardiology Solution Set Oversight Committee and Appropriate Use Criteria Task Force, American Heart Association, American Society of Echocardiography, Heart Rhythm Society, International Society for Adult Congenital Heart Disease, Society for Cardiovascular Angiography and Interventions, Society of Cardiovascular Computed Tomography, Society for Cardiovascular Magnetic Resonance, and Society of Pediatric Echocardiography. J Am Coll Cardiol.

[4] Fratz S, Chung T, Greil GF, Samyn MM, Taylor AM, Valsangiacomo Buechel ER (2013). Guidelines and protocols for cardiovascular magnetic resonance in children and adults with congenital heart disease: SCMR expert consensus group on congenital heart disease. J Cardiovasc Magn Reson.

[5] Fenchel M, Saleh R, Dinh H, Lee MH, Nael K, Krishnam M (2007). Juvenile and adult congenital heart disease: time-resolved 3D contrast-enhanced MR angiography. Radiology.

[6] Dabir D, Naehle CP, Clauberg R, Gieseke J, Schild HH, Thomas D (2012). High-resolution motion compensated MRA in patients with congenital heart disease using extracellular contrast agent at 3 Tesla. J Cardiovasc Magn Reson.

[7] Mathur M, Jones JR, Weinreb JC (2020). Gadolinium deposition and nephrogenic systemic fibrosis: a radiologist’s primer. Radiographics.

[8] Smith APL, Marino M, Roberts J, Crowder JM, Castle J, Lowery L (2017). Clearance of gadolinium from the brain with no pathologic effect after repeated administration of gadodiamide in healthy rats: an analytical and histologic study. Radiology.

[9] McDonald RJ, Levine D, Weinreb J, Kanal E, Davenport MS, Ellis JH (2018). Gadolinium retention: a research roadmap from the 2018 NIH/ACR/RSNA workshop on gadolinium chelates. Radiology.

[10] Sørensen TS, Körperich H, Greil GF, Eichhorn J, Barth P, Meyer H (2004). Operator-independent isotropic three-dimensional magnetic resonance imaging for morphology in congenital heart disease: a validation study. Circulation.

[11] Fenchel M, Greil GF, Martirosian P, Kramer U, Schick F, Claussen CD (2006). Three-dimensional morphological magnetic resonance imaging in infants and children with congenital heart disease. Pediatr Radiol.

[12] Ludwig DR, Shetty AS, Broncano J, Bhalla S, Raptis CA (2020). Magnetic resonance angiography of the thoracic vasculature: technique and applications. J Magn Reson Imaging.

[13] Edelman RR, Silvers RI, Thakrar KH, Metzl MD, Nazari J, Giri S, Koktzoglou I (2017). Nonenhanced MR angiography of the pulmonary arteries using single-shot radial quiescent-interval slice-selective (QISS): a technical feasibility study. J Cardiovasc Magn Reson.

[14] Tan EJ, Zhang S, Tirukonda P, Le Chong R (2020). REACT—a novel flow-independent non-gated non-contrast MR angiography technique using magnetization-prepared 3D non-balanced dual-echo Dixon method: preliminary clinical experience. Eur J Radiol Open.

[15] Yoneyama M, Zhang S, Hu HH, Le Chong R, Bardo D, Miller JH (2019). Free-breathing non-contrast-enhanced flow-independent MR angiography using magnetization-prepared 3D non-balanced dual-echo Dixon method: a feasibility study at 3 Tesla. Magn Reson Imaging.

[16] Isaak A, Luetkens JA, Faron A, Endler C, Mesropyan N, Katemann C (2021). Free-breathing non-contrast flow-independent cardiovascular magnetic resonance angiography using cardiac gated, magnetization-prepared 3D Dixon method: assessment of thoracic vasculature in congenital heart disease. J Cardiovasc Magn Reson.

[17] Pennig L, Wagner A, Weiss K, Lennartz S, Huntgeburth M, Hickethier T (2021). Comparison of a novel compressed SENSE accelerated 3D modified relaxation-enhanced angiography without contrast and triggering with CE-MRA in imaging of the thoracic aorta. Int J Cardiovasc Imaging.

[18] Hoyer UCI, Lennartz S, Abdullayev N, Fichter F, Jünger ST, Goertz L (2022). Imaging of the extracranial internal carotid artery in acute ischemic stroke: assessment of stenosis, plaques, and image quality using relaxation-enhanced angiography without contrast and triggering (REACT). Quant Imaging Med Surg.

[19] Dillman JR, Trout AT, Merrow AC, Moore RA, Rattan MS, Crotty EJ (2019). Non-contrast three-dimensional gradient recalled echo Dixon-based magnetic resonance angiography/venography in children. Pediatr Radiol.

[20] Eggers H, Brendel B, Duijndam A, Herigault G (2011). Dual-echo Dixon imaging with flexible choice of echo times. Magn Reson Med.

[21] Sartoretti E, Sartoretti T, Binkert C, Najafi A, Schwenk Á, Hinnen M (2019). Reduction of procedure times in routine clinical practice with compressed SENSE magnetic resonance imaging technique. PLoS ONE.

[22] Meister RL, Groth M, Jürgens JHW, Zhang S, Buhk JH, Herrmann J (2022). Compressed SENSE in pediatric brain tumor MR imaging: assessment of image quality, examination time and energy release. Clin Neuroradiol.

[23] Mesropyan N, Isaak A, Dabir D, Hart C, Faron A, Endler C (2021). Free-breathing high resolution modified Dixon steady-state angiography with compressed sensing for the assessment of the thoracic vasculature in pediatric patients with congenital heart disease. J Cardiovasc Magn Reson.

[24] Kawel-Boehm N, Hetzel SJ, Ambale-Venkatesh B, Captur G, Francois CJ, Jerosch-Herold M (2020). Reference ranges (“normal values”) for cardiovascular magnetic resonance (CMR) in adults and children: 2020 update. J Cardiovasc Magn Reson.

[25] Cicchetti DV (1994). Guidelines, criteria, and rules of thumb for evaluating normed and standardized assessment instruments in psychology. Psychol Assess.

[26] Moyé DM, Hussain T, Botnar RM, Tandon A, Greil GF, Dyer AK, Henningsson M (2018). Dual-phase whole-heart imaging using image navigation in congenital heart disease. BMC Med Imaging.

[27] Hussain T, Lossnitzer D, Bellsham-Revell H, Valverde I, Beerbaum P, Razavi R (2012). Three-dimensional dual-phase whole-heart MR imaging: clinical implications for congenital heart disease. Radiology.

[28] Valsangiacomo Buechel ER, Grosse-Wortmann L, Fratz S, Eichhorn J, Sarikouch S, Greil GF (2015). Indications for cardiovascular magnetic resonance in children with congenital and acquired heart disease: an expert consensus paper of the Imaging Working Group of the AEPC and the cardiovascular magnetic resonance section of the EACVI. Eur Heart J Cardiovasc Imaging.

[29] Hauser JA, Taylor AM, Pandya B (2017). How to image the adult patient with Fontan circulation. Circ Cardiovasc Imaging.

[30] Brown DW, Gauvreau K, Powell AJ, Lang P, Colan SD, Del Nido PJ (2007). Cardiac magnetic resonance versus routine cardiac catheterization before bidirectional glenn anastomosis in infants with functional single ventricle: a prospective randomized trial. Circulation.

[31] Pennig L, Wagner A, Weiss K, Lennartz S, Grunz J-P, Maintz D (2020). Imaging of the pulmonary vasculature in congenital heart disease without gadolinium contrast: intraindividual comparison of a novel compressed SENSE accelerated 3D modified REACT with 4D contrast-enhanced magnetic resonance angiography. J Cardiovasc Magn Reson.

[32] Kourtidou S, Jones MR, Moore RA, Tretter JT, Ollberding NJ, Crotty EJ (2019). mDixon ECG-gated 3-dimensional cardiovascular magnetic resonance angiography in patients with congenital cardiovascular disease. J Cardiovasc Magn Reson.

[33] Rathod RH, Prakash A, Powell AJ, Geva T (2010). Myocardial fibrosis identified by cardiac magnetic resonance late gadolinium enhancement is associated with adverse ventricular mechanics and ventricular tachycardia late after Fontan operation. J Am Coll Cardiol.

[34] Ghonim S, Ernst S, Keegan J, Giannakidis A, Spadotto V, Voges I (2020). Three-dimensional late gadolinium enhancement cardiovascular magnetic resonance predicts inducibility of ventricular tachycardia in adults with repaired tetralogy of fallot. Circ Arrhythm Electrophysiol.

[35] Rydman R, Gatzoulis MA, Ho SY, Ernst S, Swan L, Li W (2015). Systemic right ventricular fibrosis detected by cardiovascular magnetic resonance is associated with clinical outcome, mainly new-onset atrial arrhythmia, in patients after atrial redirection surgery for transposition of the great arteries. Circ Cardiovasc Imaging.

